# Male reproductive health statement (XIIIth international symposium on Spermatology, may 9th–12th 2018, Stockholm, Sweden

**DOI:** 10.1186/s12610-018-0077-z

**Published:** 2018-10-29

**Authors:** Hagai Levine, Hideo Mohri, Anders Ekbom, Liliana Ramos, Geoff Parker, Eduardo Roldan, Luca Jovine, Sabine Koelle, Anna Lindstrand, Simone Immler, Sharon Mortimer, David Mortimer, Gerhard van der Horst, Sumio Ishijima, Natalie Aneck-Hahn, Elisabetta Baldi, Roelof Menkveld, Susan A Rothmann, Aleksander Giwercman, Yvonne Giwercman, Mats Holmberg, Ulrik Kvist, Lars Björndahl, Rebecka Holmberg, Stefan Arver, John Flanagan, Joël R Drevet

**Affiliations:** 1 Environmental Health Track, School of Public Health, Hebrew University-Hadassah, Jerusalem, Israel; 2 University of Tokyo and National Institute for Basic Biology, Tokyo, Japan; 3 Department of Medicine, Karolinska Institute, Solna, Sweden; 4 Department of Gynaecology and Reproduction, Radboud University Medical Centre, Nijmegen, Netherlands; 5 University of Liverpool, Liverpool, UK; 6 Department of Biodiversity and Evolutionary Biology, Reproductive Ecology and Biology Group, Museo Nacional de Ciencias Naturales (CSIC), Madrid, Spain; 7 Department of Biosciences and Nutrition & Center for Innovative Medicine, Karolinska Institute, Huddinge, Sweden; 8 Anatomy & Developmental Biology, School of Medicine, Health Science Centre, University College Dublin, Belfield, Dublin, Ireland; 9 Department of Molecular Medicine and Surgery, Clinical Genetics, Center for Molecular Medicine, Karolinska University Hospital, Stockholm, Sweden; 10 School of Biological Sciences, University of East Anglia, Norwich Research Park, UK; 11 Oozoa Biomedical, Vancouver, Canada; 12 University of Sydney, Sydney, NSW Australia; 13 University of Dundee, Scotland, UK; 14 Physiology Medical School and Department of Animal Science, Stellenbosch University, National Zoological Gardens, Pretoria, South Africa; 15 School of Life Science and Technology, Tokyo Institute of Technology, Tokyo, Japan; 16 Environmental Chemical Pollution and Health Research Unit, Medical Natural Sciences (Urology), Faculty of Health Sciences, University of Pretoria, Pretoria, South Africa; 17 Department of Experimental and Clinical Medicine, University of Florence, Firenze, Italy; 18 Department of Obstetrics and Gynaecology, Faculty of Medicine and Health Sciences, Stellenbosch Universtity, Tygerberg, South Africa; 19 Fertility Solutions Inc., Cleveland, OH USA; 20 Department of Translational Medicine, Lund University, Malmö, Sweden; 21 Department of Medicine, ANOVA - Andrology, Sexual Medicine, and Transmedicine, Karolinska University Hospital, Stockholm, Sweden; 22 GReD Laboratory INSERM U1103 - CNRS UMR6293, Faculty de Medicine, CRBC, Université Clermont Auvergne (UCA), 63000 Clermont-Ferrand, France

**Keywords:** Male reproductive health, Spermatozoa, Species survival, Santé reproductive masculine, Les spermatozoïdes, Survie des espèces

## Abstract

On the occasion of the **XIIIth International Symposium on Spermatology** held from 9 to 13 May 2018 in Stockholm (Sweden), participants (guest speakers and audience) collectively felt the need to make a public statement on the general issue of male reproductive health. Our intention is to raise awareness of what we believe is a neglected area of research despite alarming situations around the world. The disclosure strategy desired by the co-authors is to bring it to the attention of the greatest number partly by considering co-publication in the various periodicals dealing with Reproductive Biology and Andrology. BaCA’s editorial office accepted this mission and found it natural that our periodical, the official journal of the French Andrology Society (SALF), should carry this message.

## Résumé

A l’occasion du **XIII**^**eme**^ **Symposium international sur la Spermatologie** qui s’est. tenu du 9 au 13 Mai 2018 à Stockholm (Suède), les participants (orateurs invités et l’auditoire) ont ressenti collectivement le besoin de faire une déclaration publique sur la question générale de la santé reproductive masculine. Notre intention est. de mieux faire connaître ce que nous pensons être un domaine de recherche négligé malgré des situations alarmantes dans le monde entier. La stratégie de divulgation souhaitée par les co-auteurs est. de le porter à l’attention du plus grand nombre en envisageant pour partie une co-publication dans les différents périodiques traitant de Reproduction et d’Andrologie. Le bureau éditorial de BaCA, a accepté cette mission et a trouvé naturel que notre périodique, journal officiel de la Société d’Andrologie en Langue Française (SALF) porte ce message.

We, scientists, public health professionals and clinicians working in spermatology, are calling on governments, organizations, the scientific and medical communities, and individuals to **acknowledge the importance of male reproductive health for the survival of the human and other species**, to **increase efforts and resources allocated to studying the causes of disruption of male reproductive health**, and to **implement policies** to remove hazards to, and promote optimal environments for, male health and reproduction.

Fertility, both female and male, and reproductive function in general are essential for the health and survival of our species. Now there is growing evidence of the effect of the male’s age and health on the offspring, that testis function is an important marker of a man’s health, and that his semen quality is a strong indicator of his future health.

Given that the sperm cell has unique qualities and a highly specific biological function, the field of spermatology continues to be a significant resource for enhancing our understanding of the most fundamental biological processes and the impact of the environment on the formation of life itself. This has potential broad implications for humans and humanity.

Until now, the field of male reproduction research has been surprisingly neglected, possibly due to cultural biases, wrongly considering reproduction to be mainly a female issue. In addition, the successful and widespread introduction of assisted reproductive technologies to circumvent even severe male factor infertility has lessened motivation to sustain research in this field as these technologies are considered to be the “remedy” for almost all causes of male infertility. However, we currently know very little about any possible long-term consequences, and so it is critical to continue research to further improve these clinical technologies and not succumb to short-cutting strategies.

A recent meta-analysis reported a steep decline in human sperm counts over the last 40 years. This decline is a source of great concern, particularly when considered alongside reports of other adverse male reproductive health trends in human and animal populations, and specific studies on hazards to male reproductive development and function.

Against this background we call on:

**Governments and organizations** to: acknowledge decreased male fertility as a major public health problem and to recognize the importance of male reproductive health for the survival of the human and other species; increase efforts and resources allocated to studying the causes of disruption of male reproduction; introduce reproductive health surveillance systems; and implement policies to prevent exposure to hazards to male fertility and ensure optimal environments for male reproductive health. Proper health promotion and education programs aimed at improving the reproductive health of populations and individuals, with specific emphasis on recognizing the crucial contribution of both sexes, are critical. There also needs to be strengthened regulatory requirements regarding the effect of pharmaceuticals on sperm function.

The **scientific and medical community** to work together to develop a global and local research agenda targeted at understanding the causes and implications of disruption of male reproductive health, and to encourage interdisciplinary research, taking one health approach and considering the developmental origin of health and diseases. We should train scientists and clinicians at all levels in andrology and in recognizing the crucial contribution of both sexes to reproduction.

**Individuals (public)** to understand that male reproductive health is critical for fecundity, that it can be evaluated, and that each man’s environment and life choices can affect his sperm quantity, quality and function, which can reflect his future health. Raising awareness may enable men to make preventive life choices and request adequate medical assessment as a part of infertility investigation.

